# Potential Mechanisms Underlying Centralized Pain and Emerging Therapeutic Interventions

**DOI:** 10.3389/fncel.2018.00035

**Published:** 2018-02-13

**Authors:** Olivia C. Eller-Smith, Andrea L. Nicol, Julie A. Christianson

**Affiliations:** ^1^Department of Anatomy and Cell Biology, University of Kansas Medical Center, Kansas City, KS, United States; ^2^Department of Anesthesiology, University of Kansas Medical Center, Kansas City, KS, United States

**Keywords:** stress, hypothalamic-pituitary-adrenal (HPA) axis, pain, exercise, cognitive behavioral therapy, central sensitization, mast cells

## Abstract

Centralized pain syndromes are associated with changes within the central nervous system that amplify peripheral input and/or generate the perception of pain in the absence of a noxious stimulus. Examples of idiopathic functional disorders that are often categorized as centralized pain syndromes include fibromyalgia, chronic pelvic pain syndromes, migraine, and temporomandibular disorder. Patients often suffer from widespread pain, associated with more than one specific syndrome, and report fatigue, mood and sleep disturbances, and poor quality of life. The high degree of symptom comorbidity and a lack of definitive underlying etiology make these syndromes notoriously difficult to treat. The main purpose of this review article is to discuss potential mechanisms of centrally-driven pain amplification and how they may contribute to increased comorbidity, poorer pain outcomes, and decreased quality of life in patients diagnosed with centralized pain syndromes, as well as discuss emerging non-pharmacological therapies that improve symptomology associated with these syndromes. Abnormal regulation and output of the hypothalamic-pituitary-adrenal (HPA) axis is commonly associated with centralized pain disorders. The HPA axis is the primary stress response system and its activation results in downstream production of cortisol and a dampening of the immune response. Patients with centralized pain syndromes often present with hyper- or hypocortisolism and evidence of altered downstream signaling from the HPA axis including increased Mast cell (MC) infiltration and activation, which can lead to sensitization of nearby nociceptive afferents. Increased peripheral input via nociceptor activation can lead to “hyperalgesic priming” and/or “wind-up” and eventually to central sensitization through long term potentiation in the central nervous system. Other evidence of central modifications has been observed through brain imaging studies of functional connectivity and magnetic resonance spectroscopy and are shown to contribute to the widespreadness of pain and poor mood in patients with fibromyalgia and chronic urological pain. Non-pharmacological therapeutics, including exercise and cognitive behavioral therapy (CBT), have shown great promise in treating symptoms of centralized pain.

## Introduction

Chronic pain, or pain lasting or recurring for more than 3 to 6 months (Merskey and Bogduck, 1994), has a high prevalence rate in the United States. There are currently 120 million chronic pain patients (Nahin, 2015), which is greater than those suffering from cardiovascular disease (85.6 million, Mozaffarian et al., 2016), diabetes (29.1 million, ADA, 2016), or cancer (14.5 million, ACS, 2016). This costs $600 billion annually due to health care costs, lost productivity, and long-term disability (Gaskin and Richard, 2012). Individuals with chronic pain may have spinal, musculoskeletal, or arthritic conditions that generate pain in a distinct and localized part of the body. Conversely, a significant proportion of patients are diagnosed with one or more specific regional or widespread pain conditions that are generally not associated with damage or disease of the affected tissue. These presumed centralized pain syndromes are generally idiopathic functional disorders with distinct adaptations within the central nervous system that amplify peripheral input and/or generate the perception of pain in the absence of peripheral input (Harper et al., 2016). Examples of centralized pain syndromes include fibromyalgia, chronic pelvic pain syndromes (irritable bowel syndrome (IBS), interstitial cystitis/painful bladder syndrome (IC/PBS), vulvodynia, and chronic prostatitis/chronic pelvic pain syndrome (CP/CPPS)), migraine, chronic fatigue syndrome (CFS), and temporomandibular disorder (Clemens et al., 2014; Clauw, 2015; Harper et al., 2016). These disorders have a high degree of co-occurrence and are generally accompanied by fatigue, sleep problems, and cognitive difficulties (Williams and Clauw, 2009). Mood disorders are also frequently encountered in patients with chronic centralized pain syndromes, including difficulty coping with stressful situations, and many suffer from depression, anxiety, and panic disorder (Arnold et al., 2006; Nickel et al., 2010; Bullones Rodríguez et al., 2013). Women are twice as likely as men to be diagnosed with a centralized pain disorder, with the obvious exception of CP/CPPS (Vincent et al., 2013). Besides sex, other factors are known to contribute to the development of centralized pain disorders including, but not limited to: abnormal neuroendocrine system and autonomic nervous system functioning, as well as environmental triggers such as psychosocial/life stressors and emotional/physical trauma (Bradley, 2008; Haviland et al., 2010).

Much debate has taken place regarding whether chronic pain states are due to “bottom up” or “top down” pain amplification mechanisms. The “bottom up” theory supports an increase in pain perception due to excess noxious peripheral input that eventually sensitizes the central nervous system to the point of perceiving pain even when there is no peripheral drive (Price and Gold, 2017). The “top down” theory suggests that changes already present within the central nervous system drive the perception of pain, regardless of peripheral noxious input (Harper et al., 2016). Regardless of mechanism, both of these theories support changes in the way the central nervous system processes noxious input and how pain is ultimately perceived. The main purpose of this review article is to discuss potential mechanisms of centrally-driven pain amplification and how they may contribute to increased comorbidity, poorer pain outcomes, and decreased quality of life in patients diagnosed with centralized pain syndromes. We highlight two phenomena that have been shown to be associated with stress-induced chronic pain disorders: dysregulation of the hypothalamic-pituitary-adrenal (HPA) axis and central sensitization. In addition, this manuscript will also explore the rodent models that are commonly employed to study the consequences of stress, which include both peripheral and central nervous system alterations, with a particular focus on chronic pelvic pain, fibromyalgia, and migraine. Finally, we describe evidence supporting exercise and cognitive behavioral therapy (CBT) as potential therapies for chronic pain disorders, including centralized pain syndromes.

## The Hypothalamic-Pituitary-Adrenal (HPA) Axis

Stress is defined as an alteration in homeostasis that can be caused by a psychological, environmental, or physiological threat (Chrousos and Gold, 1992). It has long been known to affect the perception of pain, in both acute and chronic settings. Acute stress is crucial for the survival of an organism: individuals are alerted to dangerous and life-threatening situations and can subsequently respond to the perceived or anticipated stress. However, stress can become detrimental when experienced in the long-term, especially early in life, and is associated with the development of chronic pain disorders (Anand, 1998; Bennett et al., 1998; Moore and Kennedy, 2000).

Patients with chronic pain disorders that can be partially attributed to central mechanisms, such as fibromyalgia, migraine, and chronic pelvic pain syndromes, often report a history of abuse or neglect (Hu et al., 2007; Riegel et al., 2014; Nicol et al., 2016). These patients are also more likely to present with overlapping pain syndromes and comorbid mood disorders, such as depression, anxiety, or panic disorder, with decreased quality of life scores (Nicol et al., 2016; Lai et al., 2017). One explanation for this heightened symptom severity and comorbidity is an alteration in the functioning of the HPA axis. Programming of the HPA axis happens early on in development and the perception of neglect or mistreatment can permanently affect both the regulation and output of the stress response system, as well as its downstream effects on nociceptive processing in the periphery (Heim et al., 1998; Mayson and Teichman, 2009; Burke et al., 2017).

### Central Regulation

**Figure 1:**
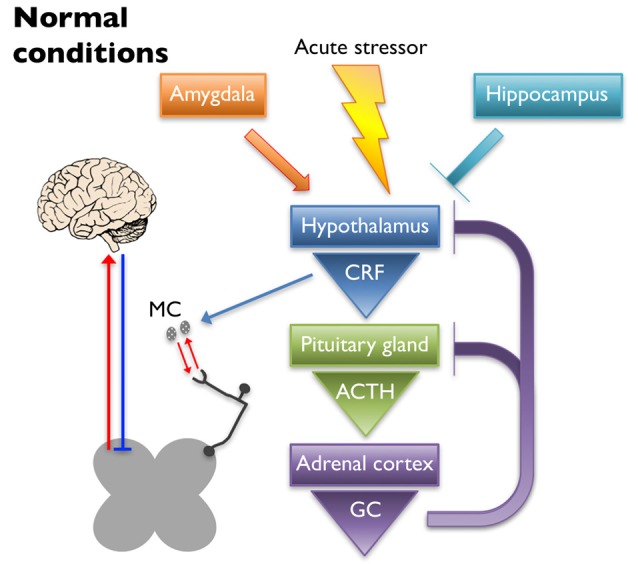
Under normal conditions, an acute stressor will signal the paraventricular nucleus (PVN) of the hypothalamus to release corticotropin-releasing factor (CRF) into the hypophyseal portal veins, which causes the anterior pituitary gland to release adrenocorticotrophic hormone (ACTH). Circulating ACTH signals the adrenal cortex to release glucocorticoids (GC) that have downstream metabolic effects. A negative feedback loop is established to turn off activation of the hypothalamic-pituitary-adrenal (HPA) axis by suppressing the production of CRF and ACTH upon cessation of the initial stressor. The hippocampus and the amygdala play inhibitory and excitatory roles in regulation of the HPA axis, respectively. CRF released upon HPA axis activation also has peripheral effects. Mast cells (MC) can become activated by CRF, causing the release of cytokines and growth factors that have reciprocal interactions with peripheral nociceptors. Nociceptor activation signals through the dorsal horn of the spinal cord, leading to activation of supraspinal somatosensory brain regions. The descending pain pathway also plays a role in the regulation of painful experiences.

The HPA axis is the primary regulator of the stress response. Under normal conditions (schematized in Figure 1), an acute stressor will signal the paraventricular nucleus (PVN) of the hypothalamus to release corticotropin-releasing factor (CRF) and arginine vasopressin into the hypophyseal portal veins, which cause the anterior pituitary gland to release adrenocorticotrophic hormone (ACTH). Circulating ACTH signals the adrenal cortex to release glucocorticoids (GCs; cortisol in humans and corticosterone in rodents) that have downstream metabolic effects (Herman et al., 2005; Ulrich-Lai and Herman, 2009). A negative feedback loop is established to turn off activation of the HPA axis by suppressing the production of CRF and ACTH upon cessation of the initial stressor (Kageyama and Suda, 2009; Tasker and Herman, 2011). Regulation of the HPA axis is driven in part by glucocorticoid and CRF receptors that are located at each level of the HPA axis and in higher limbic regions (Ulrich-Lai and Herman, 2009). Corticotropin-releasing factor receptor 1 (CRF_1_) and 2 (CRF_2_) are G-protein coupled receptors that play a prominent role in HPA axis regulation by binding CRF and its related ligands, Urocortin (Ucn) 1–3. Once activated, CRF_1_ and CRF_2_ work in opposition to one another to enhance and reduce HPA output, respectively (Bale and Vale, 2004). Glucocorticoid-mediated regulation occurs via two receptors, mineralocorticoid receptor (MR) and glucocorticoid receptor (GR) that function both as transcriptional regulators (Reul and de Kloet, 1985) and through glucocorticoid-mediated retrograde endocannabinoid release from parvocellular neurons, which suppresses the release of excitatory glutamatergic molecules from pre-synaptic terminals and subsequently inhibits the hypothalamic release of CRF (Di et al., 2003).

Limbic structures, including the hippocampus, amygdala, and prefrontal cortex, assist in resetting the HPA axis following a stressful event, as well as help regulate its tone. Neural projections from the hippocampus and prefrontal cortex are mostly glutamatergic and synapse on GABAergic interneurons within the PVN, thereby dampening HPA axis activation (Herman et al., 2003; Ulrich-Lai and Herman, 2009). Lesioning the hippocampus leads to increased stress-induced HPA axis activation as evidenced by increased CRF immunoreactivity in the PVN, glucocorticoid hypersecretion, and behavioral evidence of heightened anxiety in rats (Herman et al., 1998). Disruption of GR expression in the forebrain of mice resulted in heightened stress-induced locomotor activity and acute stress exposure increased ACTH secretion, plasma corticosterone levels, and CRF expression in the PVN (Boyle et al., 2006). The amygdala works to activate the HPA axis through disinhibition, sending GABAergic projections to the GABAergic neurons of the PVN (Herman et al., 2003; Ulrich-Lai and Herman, 2009). Administration of corticosterone to the amygdala in rats resulted in an increase in anxiety-like behaviors as well as somatic and visceral hypersensitivity (Myers and Greenwood-Van Meerveld, 2010). These observations were likely caused by GR and/or MR signaling as it was shown that repeated exposure to water avoidance stress (WAS) induced an increase in plasma corticosterone and visceral hypersensitivity that was inhibited in rats that received a GR (mifepristone) or MR (spironolactone) antagonist applied to the amygdala (Myers and Greenwood-Van Meerveld, 2012). Further evidence that the amygdala plays a role in HPA axis regulation comes from a study where either direct application of corticosterone onto the amygdala or exposure to WAS increased CRF expression in the amygdala, which coincided with visceral and somatic hypersensitivity (Johnson et al., 2015). These effects were attenuated after knock down of CRF in the central amygdala. Taken together, these studies highlight the important balance of inhibition/activation coming from the limbic structures, which plays a significant role in regulating normal stress responses from the HPA axis and ultimately affects the perception of pain.

### Downstream Signaling

While activation of the HPA axis does not directly initiate pain signaling, downstream mediators can influence the neuroimmune status of peripheral tissues and increase nociceptive tone. In human tissue, CRF_1_ has been observed in adrenal tissue, adipose, gonads, endometrium, myometrium, placenta, skin, spleen, and various immune cells; whereas CRF_2_ has been found in skin and all three types of muscle tissue (Hillhouse and Grammatopoulos, 2006). Immunoreactivity for both CRF receptors has been observed in rat colon, primarily in the mucosal layer, inflammatory cells, and enteric innervation for CRF_1_ and on goblet cells and in submucosal blood vessels for CRF_2_ (Chatzaki et al., 2004). CRF signaling influences both contractility (Buckley et al., 2014) and transepithelial resistance (Overman et al., 2012) of the gastrointestinal tract. Feline urothelial cells express functionally-active CRF_1_ and CRF_2_, as well as their intrinsic ligands CRF and Ucn1 (Hanna-Mitchell et al., 2014). The naturally-occurring feline interstitial cystitis model shows altered CRF signaling in the urothelium, indicating a potential role for CRF in the etiology of IC.

Mast cells (MCs) are a critical part of the innate immune system and are highly responsive to activation of the HPA axis, as they express five isoforms of the CRF_1_ receptor, a single isoform of the CRF_2_ receptor, and contain one of the largest peripheral stores of CRF (Theoharides et al., 2004). They are found in highly vascularized tissues, most predominantly in areas with direct contact to the environment: skin, airway, gastrointestinal and urinary tracts. They are derived from hematopoietic stem cells, circulate in the blood stream as immature cells, and mature upon entry into peripheral tissues (Kitamura, 1989; Galli and Tsai, 2010). Their differentiation depends on the presence of cytokines and growth factors. The c-kit tyrosine kinase receptor and its ligand stem cell factor (SCF) are important in the migration and distribution of MC precursors (Galli et al., 1993). MC are filled with granules that contain histamine, heparin, tryptase, as well as other proteases and cytokines (Theoharides, 1990). They are activated by immune and non-immune signals, including endogenous neuropeptides such as CRF and substance P (SP), and cause hypersensitivity reactions (Johnson and Krenger, 1992; Anand et al., 2012). Although the hallmark form of MC activation is evidenced by the partial or complete release of granular stores, stress-activated release of cytokines and growth factors from MC can occur in the absence of degranulation (Theoharides et al., 2004; Anand et al., 2012).

**Figure 2:**
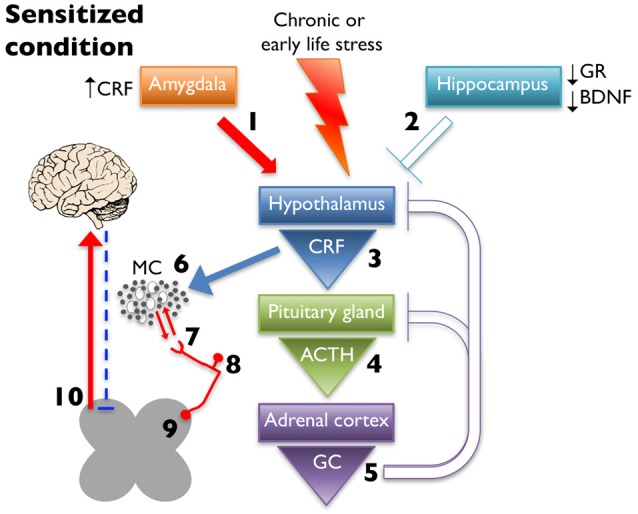
Chronic early life or adult stress leads to alteration in limbic regulation of the HPA axis. This is due to increased CRF expression and drive from the amygdala (1) and decreased glucocorticoid receptor (GR) and brain-derived neurotrophic factor (BDNF) expression in hippocampus, which dampens inhibition (2). These changes ultimately lead to increased CRF release from the hypothalamus (3), increased and prolonged release of ACTH after cessation of the stressor (4), and increased glucocorticoid (GC) production (5) with decreased negative feedback at higher structures. Increased CRF release leads to greater MC activation and infiltration (6) leading to enhanced peripheral nociceptor interaction (7). Increased peripheral drive can lead to hyperalgesic priming (8) and/or wind-up (9), eventually increasing ascending pain signaling, while simultaneously decreasing descending inhibition (10).

MCs are observed adjacent to unmyelinated nerves throughout the body, including the skin (Wiesner-Menzel et al., 1981), trachea (Uddman et al., 1985), and intestine (Stead et al., 1989) as well as in direct contact with nerve fibers in the dura mater (Rozniecki et al., 1999). These afferents express receptors involved in nociception, including transient receptor potential vanilloid 1 (TRPV1), transient receptor potential ankyrin 1 (TRPA1), and protease-activated receptor 2 (PAR2; Birder et al., 2002; Brierley et al., 2009; Kim et al., 2010). PAR2 is a G protein-coupled receptor activated by MC tryptase, trypsin, and coagulation protease FVIIa, and FXa (Ossovskaya and Bunnett, 2004). Activation of PAR2 initiates downstream sensitization of TRPV1 and TRPA1 through several mechanisms including phosphorylation by protein kinase C (PKC; Vellani et al., 2001) and protein kinase A (PKA; Bhave et al., 2003), and TRPV1 channel release from phosphatidylinositol 4,5-bisphosphate (PIP2)- dependent inhibition through phospholipase C (PLC) activation (Chuang et al., 2001). All three receptors are all expressed on neurons with cell bodies in the dorsal root ganglia (DRG), trigeminal ganglia (TG), and nodose ganglia (Steinhoff et al., 2000; Zhang and Levy, 2008; Nassini et al., 2014) and on peripheral projections to the skin and deeper tissues, such as muscle and viscera (D’Andrea et al., 1998; Bautista et al., 2013). Both TRPV1 and TRPA1 have been shown to be involved, if not required, for the generation of visceral hypersensitivity (Xu et al., 2007; Schwartz et al., 2011; DeBerry et al., 2014; Kojima et al., 2014). Increased activation of these receptors enhances pain-related afferent input to the central nervous system; however, they also generate and maintain peripheral neurogenic inflammation by releasing neuropeptides, including SP and CGRP, which perpetuates inflammatory mediator release in the proximate milieu (Julius and Basbaum, 2001). Therefore, increased peripheral CRF release due to dysregulated HPA axis activity (schematized in Figure 2) could result in MC activation and, in turn, sensitization of nearby sensory nerve endings and lowered pain thresholds.

### Role in Centralized Pain Disorders

Altered functioning of the HPA axis has been observed in patients suffering from a number of centralized pain disorders (Vierck, 2006). Approximately 20%–25% of patients with stress-related disorders have hypocortisolism, which has been postulated to come about as a compensatory response to a preceding period of hypercortisolism and excessive glucocorticoid release (Fries et al., 2005). Glucocorticoid resistance, either through reduced availability of GC or impaired function of GR, has also been proposed to contribute toward comorbidity of inflammatory disorders, including centralized pain syndromes (Silverman and Sternberg, 2012). A history of abuse or early life stress is linked to both HPA abnormalities and chronic pain syndromes; however, a clear and convincing connection between all three has yet to be fully established in a clinical setting. Conflicting studies have shown both hypercortisolism (Heim et al., 2001; Tyrka et al., 2008) and hypocortisolism (Heim et al., 1998; Gunnar and Quevedo, 2008) in adults that report a history of childhood abuse or stress. It is likely that the form of abuse and sex of the patient may influence the eventual effect on glucocorticoid production and more work is needed to determine these genetic and environmental interactions. The potential role of the HPA axis in three major centralized pain disorders, fibromyalgia, chronic pelvic pain, and migraine, is discussed in more detail below.

#### Fibromyalgia

Hypocortisolism has overwhelmingly been reported in fibromyalgia patients. A meta-analysis and meta-regression of 85 case-control comparisons reported on HPA axis involvement in functional somatic disorders, including CFS, fibromyalgia, and IBS, and showed a significant reduction in basal cortisol in all CFS and female fibromyalgia patients compared to healthy controls (Tak et al., 2011). When compared to patients with shoulder and neck pain or healthy controls, fibromyalgia patients had significantly lower waking cortisol levels (Riva et al., 2012). Strikingly, the patients with shoulder and neck pain had waking cortisol levels higher than either control or fibromyalgia patients and the authors suggest that this group may represent an intermediate step in the progression from regional to widespread musculoskeletal pain similar to the proposed mechanism of hypercortisolism leading to hypocortisolism described by Fries et al. (2005). A recent study compared basal and stress-evoked salivary cortisol levels between fibromyalgia and control patients with and without a history of early childhood abuse (Coppens et al., 2017). They reported a decrease in stress-evoked cortisol release in fibromyalgia patients, that was largely driven by increased cortisol release in control patients with a history of early childhood abuse. This observation again underscores the disparate effects of childhood experiences on later output of the HPA axis and its involvement in pain processing.

Rodent models of adult stress are commonly used to induce behaviors and molecular changes similar to what is observed in fibromyalgia patients. These models include intermittent cold stress (Nishiyori and Ueda, 2008), unpredictable sound stress (Green et al., 2011), WAS (Chen et al., 2011), and restraint stress (Bardin et al., 2009) and induce long-lasting widespread mechanical allodynia and hyperalgesia. An early life stress model of fibromyalgia that incorporates limited nesting material during the pre-weaning period produces adult rats that display mild muscle hyperalgesia that worsens following sound stress (Alvarez et al., 2013). We have reported hind paw mechanical hypersensitivity in both male (Fuentes et al., 2016) and female (Pierce et al., 2014) mice that also demonstrate urogenital hypersensitivity (discussed below) following neonatal maternal separation (NMS). Interestingly, we observed evidence of decreased HPA axis output in male (Fuentes et al., 2018) and increased HPA axis output in female (Pierce et al., 2016) NMS mice, suggesting that regardless the effect of early life stress on glucocorticoid production, widespread allodynia is a common final pathway of HPA axis dysregulation in this model.

The most commonly employed rodent models of fibromyalgia involve direct activation of the peripheral nervous system via intramuscular injection of carrageenan (Kehl et al., 2000) or acidic saline (Sluka et al., 2001). Carrageenan is a chemical nociceptive stimulus that evokes inflammation and excites muscle nociceptors (Hargreaves et al., 1988). When injected intramuscularly, grip strength is reduced in the ipsilateral limb (Kehl et al., 2000). Repeated intramuscular injection of low pH saline causes ipsilateral and contralateral mechanical hyperalgesia lasting 4 weeks after the second injection (Sluka et al., 2001), likely via activation of acid-sensing ion channels (ASIC) present on primary afferent fibers (Waldmann and Lazdunski, 1998). The use of these stress- or nociceptor-induced models can be used to tease out the various “bottom up” and “top down” mechanisms that likely contribute to the disparate etiologies underlying fibromyalgia (Sluka and Clauw, 2016).

#### Chronic Pelvic Pain

Alterations in HPA axis output have been reported for all chronic pelvic pain syndromes, although the impact, in terms of hyper- or hypocortisolism, largely depends on the type of syndrome. Patients with IBS have increased basal and evoked cortisol release that, in some cases, is linked to early life adverse events (Chang et al., 2009; Videlock et al., 2009). Treatment with CRF_1_ antagonist has shown mixed clinical results with no effect on stooling symptoms in diarrhea-predominant IBS patients (Sweetser et al., 2009), but a positive impact on significantly reducing the blood oxygen level-dependent signal in the hypothalamus in IBS patients (with average or high levels of anxiety) during the expectation of abdominal pain (Hubbard et al., 2011). Men with CP/CPPS have greater waking cortisol levels (Anderson et al., 2008) and delayed ACTH release in response to an acute stressor, which correlates with significant psychological disturbances (Anderson et al., 2009). Finally, women with vulvodynia have blunted serum cortisol cycles and reported higher symptoms of stress compared to healthy controls (Ehrstrom et al., 2009).

Despite showing disparate cortisol levels, chronic pelvic pain syndromes all have evidence of increased activation downstream of the HPA axis in affected peripheral tissues. Biopsies from patients with IBS (Barbara et al., 2004; Akbar et al., 2008), CP/CPPS (Theoharides et al., 1990; Amir et al., 1998), IC/PBS (Larsen et al., 2008; Liu et al., 2012), and vulvodynia (Leclair et al., 2011) all revealed increased MC infiltration and altered granular structure including a reduced proportion of intact MC. Serum (Jiang et al., 2013) and urine (Corcoran et al., 2013) samples from IC/PBS patients also had elevated MC granule components, including nerve growth factor (NGF), histamine, and pro-inflammatory cytokines, indicating an increase in MC activation. Tissue biopsies from IC/PBS patients revealed increased MC infiltration in close proximity to densely-populated SP-immunopositive nerve fibers (Pang et al., 1995). Expressed prostatic secretions from CP/CPPS patients had elevated MC tryptase and NGF levels (Done et al., 2012) and urine samples also had increased tryptase, as well as carboxypeptidase A3, a marker of MC activation (Roman et al., 2014).

In line with these observations in humans, MC activation, histamine release, NGF expression, and associated pelvic organ hypersensitivity have all been shown to be increased by stress exposure in adult male rats (Merrill et al., 2013). We demonstrated that NMS increased vaginal (Pierce et al., 2014), bladder (Pierce et al., 2016), and referred prostatic (Fuentes et al., 2015, 2018) sensitivity in mice, which corresponded to an increased percentage of degranulated MC in urogenital tissues, compared to naïve mice. Exposure to WAS further increased MC degranulation in the bladder of NMS and naïve female mice (Pierce et al., 2016) and the prostate of NMS and naïve male mice (Fuentes et al., 2018). The limited nesting material method was also employed for assessing visceral sensitivity. Adult male rats that were exposed to limited nesting material as neonates exhibited increased colonic sensitivity and anxiety behaviors, which were not present in female littermates (Prusator and Greenwood-Van Meerveld, 2015). The finding is particularly intriguing considering the larger number of female chronic pelvic pain patients, as well as the preponderance of preclinical research that has been done in male rodents. Both restraint stress and central administration of CRF caused MC degranulation in the colon in rats (Pothoulakis et al., 1998). The role of MC activation in stress-induced pain is further illustrated by studies using MC stabilizers. When injected 30 min before restraint stress, the MC stabilizer doxantrazole attenuated the stress-induced increase in abdominal contractions during CRD (Gué et al., 1997). Pre-treatment with a non-specific CRF antagonist, α-helical-CRF, also prevented stress-induced visceral hypersensitivity in NMS rats following WAS, in a MC-dependent manner (van den Wijngaard et al., 2012). Treatment with a MC stabilizer, but not α-helical-CRF, was also capable of reversing WAS-induced visceral hypersensitivity, suggesting that non-CRF dependent factors are involved in the maintenance of post-stress hypersensitivity. In a study of a transgenic autoimmune cystitis mouse model of IC/PBS that displays bladder inflammation, increased number of MC, and urinary tract dysfunction, treatment with the MC stabilizer cromolyn reversed these symptoms (Wang et al., 2016). Furthermore, crossing this transgenic model with MC-deficient mice produced mice with reduced bladder inflammation and no urinary tract dysfunction. However, both of these symptoms were reestablished upon MC reconstitution.

#### Migraine

Stress is a commonly-reported trigger for migraine attack; however, evidence of altered cortisol release, compared to other centralized pain syndromes, is less prevalent among migraine patients. A recent literature review of seven cross-sectional studies largely showed no baseline differences in cortisol level between migraineurs and healthy controls (Lippi and Mattiuzzi, 2017). A potential for increased HPA axis responsiveness was noted, as nitroglycerin- and CRF-evoked cortisol levels were reportedly higher in migraineurs, compared to healthy controls, across four observational studies. Another study looking at blood cortisol levels over a 12-h period revealed a greater total cortisol release and peak in migraineurs compared to control patients (Wang et al., 2016). One prospective study of 17 migraine patients reported on stress-related parameters prior to and during a migraine attack (Schoonman et al., 2007). While a subgroup of patients did report an increase in perceived stress, which also reportedly triggered their migraine attacks, no objective measures of increased cortisol or other measures of a biological stress response were present either prior to or during a migraine attack. The mouse model of Familial Hemiplegic Migraine type 1 (FHM1), which was generated by knocking-in an R192Q missense Ca_v_2.1 Ca^2+^ channel mutation, displayed an increase in cortical spreading depression following treatment with corticosterone, which was blocked by pretreatment with a GR antagonist (Shyti et al., 2015). The same FHM1 mice did not show increased cortical spreading depression following an acute stress. The authors explained this discrepancy as a direct effect of corticosterone on glutamatergic neurotransmission, via a GR-mediated mechanism, that is otherwise counteracted during bouts of acute stress. This observation suggests that future studies on the effect of stress and the HPA axis on migraine should take into account the type of stressor and etiology of the patient, as migraineurs represent a heterogeneous population of patients.

It has been known for several decades that patients with migraine have increased plasma histamine levels, an indicator of MC activation, both during migraine attack and at rest, when compared to healthy controls (Heatley et al., 1982). MC reside in the dura and have been hypothesized to release their pro-inflammatory contents following activation by neuropeptides released from nearby sensory nerve endings, thereby generating neurogenic inflammation (Levy, 2009). A recent study revealed that cultured human MCs do not express receptors for either calcitonin gene-related peptide (CGRP) or pituitary adenylate cyclase-activating polypeptide (PACAP), the two neuropeptides most commonly associated with migraine, but rather express and release PACAP upon activation (Okragly et al., 2017). Animal models of migraine have investigated the role of MCs more thoroughly and shown that application of MC mediators can sensitize dural afferents and evoke migraine-like behaviors (Yan et al., 2013). Restraint stress has also been shown to induce dural MC degranulation and increase protease I levels in the cerebrospinal fluid (Theoharides et al., 1995). These outcomes could be attenuated by pretreatment with antisera to CRF or neonatal treatment with capsaicin to deplete peptidergic innervation, further supporting the role of neuropeptide release in MC activation and the generation of migraine.

## Central Sensitization

Central sensitization is defined as “an amplification of neural signaling within the central nervous system that elicits pain hypersensitivity” (Woolf, 2011). It presents as allodynia, a painful response to a stimulus that is usually considered “non-noxious”, and/or hyperalgesia, an increased response to a noxious stimulus, in widespread locations in addition to areas associated with the underlying pain disorder (Woolf, 2011).The mechanism underlying central sensitization is not fully understood, but it is likely that there are both peripheral and central components that play a role in the establishment and maintenance of this phenomenon that is seen in many centralized pain disorders. The instigating factor of central sensitization could originate in the periphery through mechanisms that eventually lead to long-term potentiation (LTP) in the spinal cord as well as structural changes in the brain. These central nervous system changes are then key to the maintenance of increased pain perception.

### “Hyperalgesic Priming” and “Wind Up”

Both peripheral and central neurons show great plasticity, meaning they are able to adapt to the sensory information that they receive. This usually leads to an amplification of signaling (Woolf and Walters, 1991) and can establish a “pain memory” (Dennis and Melzack, 1979). One such “pain memory” is termed “hyperalgesic priming”, which occurs when a peripheral nociceptor is exposed to an injury or other priming event that results in long-term alterations rendering the afferent more excitable to subsequent activation (Reichling and Levine, 2009). One proposed mechanism behind hyperalgesic priming is increased localized translation of mRNA that is otherwise kept in a dormant state at the synapse (Price and Geranton, 2009). This rapid response in translation is made possible by ribosomes located at the base of dendritic spines (Steward and Levy, 1982), avoiding the need to traffic mRNA from the cell body to effect changes in gene expression. NGF and IL-6 are able to activate kinases important for protein synthesis including the mechanistic target of rapamycin complex 1 (mTORC1) and extracellular signal regulated kinase (ERK). An increase in mTORC1 and ERK signaling at the synapse leads to local increase in protein synthesis (Melemedjian et al., 2010) and therefore alterations in nociceptor sensitivity. Once this “pain memory” is established in the nociceptor, it can eventually become established in the CNS as a form of LTP. Although most spinal LTP is thought to be induced by high-frequency stimulation of afferent fibers, it has been shown that activation of low frequency c-fibers can lead to LTP in the spinal dorsal horn (Ikeda et al., 2006).

Another process involved in the development of central sensitization is known as “wind-up”, which involves repetitive low frequency input to peripheral c-fibers leading to temporal summation and activation, ultimately generating a pain response (Mendell and Wall, 1965). This leads to hypersensitivity characterized by lowered thresholds necessary to elicit and maintain wind-up (Li et al., 1999). Wind-up is commonly seen in fibromyalgia patients and, once it is maintained, pain ratings to subsequent stimuli are higher and more prolonged than those of healthy control patients (Staud et al., 2004). Trigeminal wide dynamic range neurons also display wind-up and may play a role in chronic pain in the orofacial region including migraine and TMD (Coste et al., 2008).

NGF has been implicated in “hyperalgesic priming” as a mediator of synaptic protein synthesis (Price and Inyang, 2015), whereas SP-binding and internalization of its receptor, Neurokinin-1 (NK1), is necessary for sensitization of dorsal horn neurons and LTP (Ikeda et al., 2006). MC are a significant source of both NGF and SP (Leon et al., 1994) and, as discussed in the previous section, are activated and increased in a number of centralized pain disorders (Theoharides et al., 2001, 2005; Lucas et al., 2006; Walker et al., 2011). Additionally, increased NGF and SP levels have been detected in the CSF of patients with fibromyalgia (Russell et al., 1994; Giovengo et al., 1999), chronic daily headache (Sarchielli et al., 2001), and CP/CPPS (Miller et al., 2002). An increase in SP-immunopositive nerves has been also observed in bladders of IC patients (Pang et al., 1995) and SP release activates TRP channels, resulting in hypersensitivity in rodent models of chronic pelvic pain (Wick et al., 2006; Pan et al., 2010). Therefore, NGF and SP are uniquely poised to play roles in hypersensitivity resulting from HPA axis abnormalities and in the establishment of central sensitization.

### Functional and Structural Changes within the Brain

Increased peripheral input eventually results in long-term or permanent changes within the brain, particularly in regions associated with the affective component of pain. Repetitive activation of peripheral nociceptors causes remodeling of the central nucleus of the amygdala (CeA; Cheng et al., 2011), which is important in pain regulation and the emotional response to pain. Additionally, cortical plasticity and LTP has been reported following peripheral nerve injury (Zhuo, 2008). Specifically, changes in structure and connectivity are seen in the anterior cingulate cortex (ACC) and insular cortex (IC) of chronic pain patients (Kutch et al., 2017b). Alterations in these brain regions can lead to diminished descending inhibitory control and facilitatory pain signaling through their connections that ultimately terminate in the spinal cord. Two other major components of the descending pain inhibitory pathway, the periaqueductal gray (PAG) and brainstem rostral ventromedial medulla (RVM), project to inhibitory interneurons in the spinal cord and turn off nociceptive signals under normal conditions (Zhuo, 2008). Decreased function of these inhibitory interneurons is yet another mechanism that could be underlying central sensitization (Scholz et al., 2005).

Recent advances in brain imaging techniques have allowed for thorough examination of gray matter volume, functional connectivity, and metabolite levels in pain-relevant areas of the brain in chronic pain patients. While brain imaging studies of chronic back pain patients have revealed decreased gray matter in areas involved with pain perception and modulation (Apkarian et al., 2004), patients with centralized pain syndromes largely show an increase in gray matter that is associated with greater widespread pain and comorbidity (Schmidt-Wilcke et al., 2007; Schweinhardt et al., 2008; Seminowicz et al., 2010; Kutch et al., 2017a). Functional connectivity between sensorimotor and insular cortices have largely been reported in chronic urological pain patients, again with an association with pain that is more widespread and lower quality of life scores (Kutch et al., 2015, 2017b; Harper et al., 2018). Abnormal levels of choline and GABA have been observed in the ACC of chronic urological pain patients, which was associated with greater functional connectivity and negative mood (Harper et al., 2018).

### Evidence of Central Sensitization in Centralized Pain Syndromes

Fibromyalgia is characterized by central sensitization due to widespread musculoskeletal pain, hypersensitivity to normally non-noxious stimulation of tissue (allodynia), and physical and mental fatigue associated with the disorder (Mease et al., 2005). Fibromyalgia patients display reduced mechanical and thermal pain thresholds in the absence of tissue injury (Desmeules et al., 2003; Petzke et al., 2003), as well as a decrease in the frequency of stimulation needed to maintain experimentally-provoked pain (Staud et al., 2004). Likewise, the aforementioned brain imaging studies support long-term structural and functional changes within the brains of patients with fibromyalgia (Cummiford et al., 2016; Kutch et al., 2017a).

Central sensitization is also believed to play a role in migraine due to the wide range of symptoms that present in this disorder including throbbing cranial pain, sensitivity to light (photophobia) and sound (phonophobia), nausea, fatigue, irritability, muscle tenderness, and cutaneous allodynia including extracephalic allodynia (Silberstein, 1995). Studies have found that rats display both local (facial) and widespread (hind paw) allodynia following meningeal application of noxious agonists to elicit migraine (Wieseler et al., 2010; Burgos-Vega et al., 2016). The pathway involved in migraine, the trigeminovascular pathway, is made up of central neurons located in the spinal trigeminal nucleus that receive sensory input from the meninges, periorbital skin, and pericranial muscles and subsequently send projections to the thalamus leading to pain perception (Burstein et al., 2000). Sensitization of the trigeminovascular pathway is thought to not only underlie the coinciding headache pain and cutaneous allodynia experienced during migraine, but also the association with widespread dysregulation of pain perception and, consequently, the presentation of comorbid chronic pain disorders. In a large study of migraine patients, it was shown that migraineurs with allodynia were more likely to be diagnosed with depression, IBS, fibromyalgia, or CFS than those without cutaneous allodynia (Tietjen et al., 2009). Additionally, there was a significant positive correlation in the number of pain disorders experienced and the severity of allodynic symptoms.

Central sensitization could also play a role in the establishment and maintenance of chronic pelvic pain. For example, IC/PBS, a disorder characterized by bladder and pelvic pain and an increase in urinary voidance frequency (Hanno et al., 2011), has been found to be associated with other pelvic pain disorders as well as disorders beyond the pelvis including fibromyalgia and migraine (Warren et al., 2011). Clauw et al. (1997) investigated the association of IC/PBS and fibromyalgia and found that both fibromyalgia and IC/PBS patients exhibited greater tenderness than controls at all points measured, with fibromyalgia patients displaying greater responses to tender points. This indicates that similar to fibromyalgia patients, IC/PBS patients also display widespread allodynia suggesting a dysfunction in the CNS pain processing pathways in both of these disorders.

## Therapeutic Interventions

A growing area of research in the study of pain is non-pharmacological therapeutic interventions for treating chronic pain disorders. These therapies include exercise, CBT, trigger point injection, physical therapy, and neuromodulation (Till et al., 2017). The advantage of these therapeutic interventions is that they generally result in symptom improvement without the harmful side effects commonly associated with pharmacological treatments (Table 1).

**Table 1:** Evidence of non-pharmacological therapies for the treatment of centralized pain symptoms and associated comorbidities.

Therapeutic treatment	Disorder	Outcomes measured	References
Exercise (walking, aerobic strength training, yoga, pilates, or swimming)	Psychological disorders	Depression scores; anxiety scores; mood	Byrne and Byrne (1993)
	Irritable bowel syndrome	Irritable bowel specific quality of life, GI symptoms (constipation, diarrhea, pain)	Daley et al. (2008) and Johannesson et al. (2011)
	Chronic prostatitis and/or chronic pelvic pain	Pain scores; quality of life	Dhillon and Holt (2003), Zhang et al. (2015) and Saxena et al. (2017)
	Migraine	Headache frequency, headache intensity, number of headache days, disability, quality of life, depression, anxiety	Kelman (2007), Baillie et al. (2014), Santiago et al. (2014) and Daenen et al. (2015)
	Fibromyalgia	Fibromyalgia Impact Questionnaire score, 6-min walk test, self-efficacy, grip strength, pain severity, social functioning, quality of life, psychological distress, brain response and pain rating to heat stimuli	Gowans et al. (1999), Mannerkorpi et al. (2000, 2010), Busch et al. (2011), Thompson (2012) and Ellingson et al. (2016)
Cognitive behavioral therapy	Psychological disorders	Depression scores, self-esteem scores, anxiety scores	Reynolds and Coats (1986), Lewinsohn and Clarke (1999), Hofmann and Smits (2008)
	Chronic pelvic pain	Pelvic pain, widespread pain, dyschezia, dyspareunia, quality of life, disability, depression, anxiety	Eccleston et al. (2014) and Meissner et al. (2016)
	Migraine	Headache frequency, headache duration, headache intensity, anxiety, depression, self-efficacy	Andrasik (2007)
	Fibromyalgia	Fibromyalgia Impact Questionnaire score, 6-min walk test, self-efficacy, quality of life, social functioning, psychological distress, McGill ratings of pain, physical functioning	Gowans et al. (1999), Mannerkorpi et al. (2000) and Williams et al. (2002)

### Exercise

Exercise is defined as physical activities that are planned, structured, repetitive, and centered on an improvement in physical health (Caspersen et al., 1985). In humans, exercise therapy can take the form of a wide range of activities such as walking, aerobic strength training, yoga, pilates, or swimming. This variety is important so that a patient can find an activity that they enjoy and therefore are more likely to view exercise as a long-term life style change rather than a quick fix treatment. Exercise has been shown to relieve stress and reduce depression and anxiety (Byrne and Byrne, 1993) and has successfully been implemented in the treatment of chronic pain disorders including chronic pelvic pain (Daley et al., 2008), migraine (Baillie et al., 2014), and fibromyalgia (Thompson, 2012). In a 12-week exercise intervention in patients with IBS, exercise led to improvements in the IBS Severity Scoring System score, which includes measurements in pain severity and pain frequency, and IBS-quality of life score, which measures qualities related to emotional functioning compared to patients that received usual care for IBS (Johannesson et al., 2011). Similarly, in a cohort of women with chronic pelvic pain, an 8-week yoga intervention resulted in improvement in pain and quality of life scores compared to the control group that was treated with non-steroidal anti-inflammatory drugs (Saxena et al., 2017). The frequency, intensity, and duration of migraine pain was significantly reduced when 12 weeks of outdoor walking was added to the treatment regime of migraine patients taking amitriptyline (Santiago et al., 2014) and Nordic walking (walking with poles) for 15 weeks resulted in improvements in the Fibromyalgia Impact Questionnaire score compared to control fibromyalgia patients (Mannerkorpi et al., 2010). Not only has exercise been utilized as a therapeutic intervention for chronic pain, being more physically active has also been shown to reduce the chance of developing chronic pain disorders. Specifically, higher levels of both moderate- and high-intensity physical activity were associated with a lower risk of developing CP/CPPS in older men (Zhang et al., 2015) and endometriosis in young adult women (Dhillon and Holt, 2003).

In rodents, exercise has been shown to have positive effects on neurodevelopment (van Praag et al., 1999), as well as increase neuronal survival and resistance to brain insult (Carro et al., 2001), and stimulate brain vascularization (Black et al., 1990). Exercise protocols can take the form of resistance training or aerobic exercise such as wheel running, treadmill running, and swimming. Voluntary wheel running prevented the development of autonomic dysfunction and paw and muscle allodynia in a mouse model of fibromyalgia (Sabharwal et al., 2016). Forced treadmill running in rodents could be considered a stressor due to the fact that it generally involves the use of an aversive stimulus, such as probing or foot shock, to provoke the rodent to continue running. In contrast, voluntary wheel running is considered rewarding, as most rodents will choose to run when provided a running wheel (Sherwin, 1998) and therefore this form of exercise is generally not viewed as a stressor. In support of this, corticosterone levels and anxiety behaviors have been shown to be elevated after forced treadmill running compared to sedentary controls, but these effects were not seen in rodents following voluntary wheel running (Leasure and Jones, 2008; Ke et al., 2011; Svensson et al., 2016). Furthermore, voluntary exercise induced higher hippocampal brain-derived neurotrophic factor (BDNF) concentration compared to rats subjected to forced exercise or sedentary controls (Ke et al., 2011).

**Figure 3:**
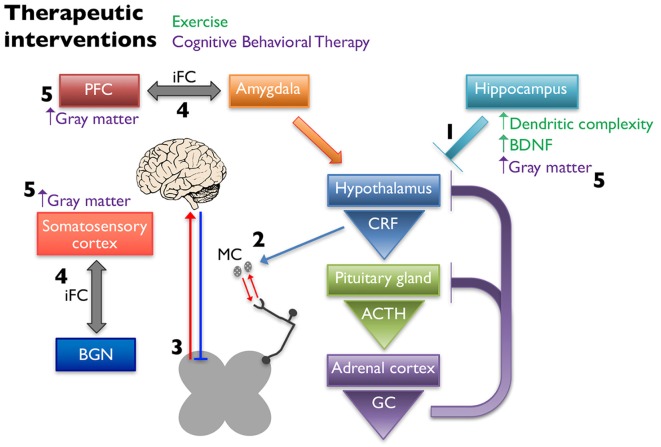
Non-pharmaceutical interventions restore proper signaling within the HPA axis and between higher structures. Exercise increases dendritic complexity and BDNF expression in the hippocampus, which restores negative input onto the hypothalamus to restore proper HPA axis output (1). Decreased CRF release stabilizes MC activation and infiltration associated with chronic pain disorders (2), thereby reducing peripheral nociceptive input. Exercise influences the descending pain pathway, likely through release of endogenous opioids, increasing neuronal activity, and balancing excitatory and inhibitory transmission (3). Cognitive behavioral therapy (CBT) alters the intrinsic functional connectivity (iFC) between brain regions associated with pain management, including connections between the prefontal cortex (PFC) and amygdala and somatosensory cortex and the basal ganglia network (BGN) (4). Cortical and hippocampal gray matter densities are also increased in patients following CBT (5).

A potential explanation for the beneficial effects of exercise is that it influences gene expression and structural complexity in the limbic structures that regulate the HPA axis (Figure 3). Specifically, running wheel access has been shown to normalize hippocampal GR and BDNF mRNA levels in NMS rats (Maniam and Morris, 2010), and increase neurogenesis and dendritic spine density in the hippocampus of adult rats (Stranahan et al., 2007). Other groups have also evaluated the effects of exercise on stress-induced changes in rodents. It was shown that chronic unpredictable stress induced a depressive phenotype in rats, decreased hippocampal BDNF and GR mRNA levels, and increased circulating corticosterone, while 4 weeks of voluntary wheel running attenuated these effects (Zheng et al., 2006). Similarly, 3 weeks of voluntary wheel running before exposure to immobilization stress was able to prevent the decrease in BDNF protein levels caused by the stressor (Adlard and Cotman, 2004). These findings are relevant to the study of treatment of chronic pain because of the association of stress causing or exacerbating pain symptoms as previously described.

Another hypothesis for the mechanism underlying the positive influence of exercise on pain is through improved conditioned pain modulation (CPM), formerly referred to as diffuse noxious inhibitory control (DNIC). CPM is a “pain-inhibits-pain” mechanism and represents a measure of the function of descending analgesic pathways. This system has been shown to be defective in chronic pain patients (Lewis et al., 2012) and these deficits in pain inhibitory pathways are involved in the development of central sensitization (Scholz et al., 2005). The relationship between exercise and improved CPM was demonstrated in a study evaluating the ability of self-reported physical activity to predict thermal sensitivity as well as pain facilitatory and inhibitory function, tested by temporal summation (TS; perceived increase in pain intensity to repeated stimulation at a constant stimulus intensity, reflecting central sensitization) and CPM respectively. Results indicated that individuals reporting greater total physical activity showed reduced TS of pain and greater CPM (Naugle and Riley, 2014).

There are different mechanisms that could explain the beneficial influence of exercise on CPM including causing an increase in endogenous opioids, stimulating brain structures involved in descending analgesic pathways, and/or maintaining the balance between excitatory (glutamate) and inhibitory (GABA) neurotransmitters in the CNS (Naugle and Riley, 2014). Exercise increases endogenous opioids in the CNS (aan het Rot et al., 2009) and the effects of this increase are often compared to effects following administration of the opioid receptor antagonist naloxone. One study assessing changes in pain threshold after swimming found that there was a significant increase in hind limb hot plate withdrawal threshold in exercised mice that received saline injection before exercise but no increase in pain threshold in exercised mice that received naloxone (Willow et al., 1980). Similar results were seen in humans when saline or naloxone was administered to long-distance runners after a run. Long-distance running produced thermal and ischemic hypoalgesia and mood elevation, but most of these effects were attenuated after administration of naloxone (Janal et al., 1984). Further evidence of the influence of exercise on CPM comes from studies evaluating brain activity in areas involved in descending pain modulation pathways. Ellingson et al. (2016) examined brain activity during administration of painful heat stimuli following moderate intensity cycling exercise or quiet rest in fibromyalgia patients. They found that exercise, but not quiet rest, prior to heat stimulation, elicited increased activity in brain regions involved in the anterior insula and dorsolateral prefrontal cortex, as well as lower pain ratings. A final potential mechanism regarding the beneficial role of exercise on CPM is that exercise can influence the balance of excitatory and inhibitory transmission in pain pathways. Four weeks of voluntary wheel running in rats resulted in significant changes in the forebrain GABAergic system compared to sedentary control rats (Hill et al., 2010). All of the previously described results suggest that exercise significantly alters structures and signals involved in CPM and these alterations could begin to explain why exercise has been shown to reduce pain symptoms in many chronic pain disorders.

It is important to note that the length of exercise protocol, as well as intensity of exercise, have been shown to have differential outcomes. In regards to duration of exercise intervention, both short- and long-term periods of voluntary wheel running increased cell proliferation in the rat hippocampus, but a longer-term exercise protocol was required to increase neurogenesis (14 days) and LTP (56 days; Patten et al., 2013). In a study comparing the effects of low-intensity running, high-intensity running, or sedentary conditions in rats, it was found that low-intensity running increased BDNF levels and dendritic complexity and branching in the hippocampus while not significantly affecting corticosterone levels. In contrast, high-intensity exercise did not increase hippocampal BDNF levels or induce structural hippocampal changes but did cause a significant increase in corticosterone. This suggests that the high-intensity exercise elicited a stressful response and therefore did not have the beneficial effects that the low-intensity exercise did (Shih et al., 2013). These preclinical results are similar to what is seen in humans, where there appears to be a narrow “therapeutic window” for the use of exercise in chronic pain treatment. For example, the majority of studies evaluating the efficacy of exercise for fibromyalgia treatment have found that low or moderate levels of aerobic training reduces tender point pain compared to before training or a control group. However, more strenuous exercise regimes often result in an increase in fibromyalgia pain (Vierck, 2006). Additionally, it has been shown that the greatest improvement in fibromyalgia patients is seen after long-term (12 weeks) moderate intensity aerobic training (Busch et al., 2011). There is evidence that exercise can be a trigger or increase pain during migraine attacks (Kelman, 2007). However, it is suggested that exercise treatment for migraine be implemented with a slow increase in intensity and duration to allow the patient to habituate to exercise and eventually see benefits (Daenen et al., 2015). Therefore, in the instances where exercise has been found to have detrimental effects on chronic pain symptoms, it is likely due to improper application of exercise treatment. This highlights the importance of tailoring exercise regimes specifically to individual patients to achieve the most benefit and avoid exacerbation of pain symptoms due to overly intense exercise prescription.

### Cognitive Behavioral Therapy

Another form of non-pharmacological therapy is CBT, which consists of educating patients about their pain, techniques on how to cope with their pain, such as relaxation training, and how to implement these cognitive coping techniques in real-life situations (Waters et al., 2007). This technique is particularly useful in stress-induced pain syndromes as the premise behind CBT is to reduce stress and emotional responses and thereby subsequently reduce symptom severity or frequency. The use of CBT for improving symptoms of depression and anxiety has been well-established (Reynolds and Coats, 1986; Lewinsohn and Clarke, 1999; Hofmann and Smits, 2008). In a study evaluating CBT as a form of fibromyalgia treatment, a greater percentage of patients displayed improvement in physical functioning after CBT compared to patients that received standard care in the form of pharmacological management of symptoms (Williams et al., 2002). Although CBT is most commonly used in treating symptoms in fibromyalgia patients, it has also been used effectively to treat chronic pelvic pain symptoms. In a study of women with endometriosis-associated pelvic pain, CBT was implemented in addition to somatosensory stimulation in the form of acupuncture and this combination of treatments resulted in a decrease in pelvic pain and an increase in quality of life (Meissner et al., 2016). Additionally, a recent review categorized studies that used Internet-delivered CBT into headache and non-headache chronic pain conditions. They found that pain, disability, depression, and anxiety were reduced in non-headache chronic pain conditions and there was insufficient evidence to make conclusions in headache conditions due to having only two studies to analyze (Eccleston et al., 2014). However, another review focused on CBT in headache disorders found that CBT reduced headache activity 30%–60% on average across studies but notes that there are a fair number of patients who were non-responders (40%–70%; Andrasik, 2007).

It is hypothesized that the positive effects of CBT seen in the treatment of chronic pain are due to structural changes in the gray matter in regions of the brain associated with pain management and/or in the functional connectivity of these regions (Figure 3). CBT induced changes in gray matter volume have been observed when implemented to treat patients with chronic pain disorders (Seminowicz et al., 2013), CFS (de Lange et al., 2008), and posttraumatic stress disorder (Levy-Gigi et al., 2013). One study found that 11 weeks of CBT in a mixed group of chronic pain patients resulted in significant gray matter differences in sensory, motor, and affective brain areas as measured using voxel-based morphometry (Seminowicz et al., 2013). The majority of the CBT induced changes were increases in gray matter, however one region, the supplementary motor area (SMA), showed a significant gray matter decrease after CBT. The patients in this study showed significant improvements in pain measurements as well as region-specific positive and negative correlations in gray matter changes and pain catastrophizing. Changes in intrinsic functional connectivity (iFC) are also seen in areas of the brains of chronic pain patients. Baliki et al. (2012) found that functional connectivity patterns between the medial prefrontal cortex and nucleus accumbens could predict whether a patient with sub-acute back pain would recover or develop persistent pain. They also describe iFC differences in other brain regions including the insula and basal ganglia in these patients (Baliki et al., 2012). In a study investigating the effect of CBT on iFC, 11-weeks of CBT in patients with various types of chronic musculoskeletal pain led to significant pre-post changes in iFC. Specifically, they found a decrease in iFC between the medial prefrontal cortex and amygdala and PAG and an increase in iFC between the basal ganglia network (BGN) and the right secondary somatosensory cortex. These changes were correlated with significant improvements in clinical measures of pain. CBT patients that showed the greatest improvement in self-efficacy and pain symptoms exhibited the greatest pre-post change in iFC. These results were not seen in an active control group who received educational materials that did not include CBT (Shpaner et al., 2014).

Due to the fact that no treatment for chronic pain disorders has been found to be universally successful, it is imperative to try and find alternative treatments to those that are currently available. Additionally, due to the negative side effects that often accompany pharmacological therapies, finding successful treatments that avoid these side effects is key. Evidence suggests that exercise and CBT are both able to improve symptoms associated with multiple chronic pain disorders, usually without harmful side effects. It is likely that a combination of therapies tailored specially to individual patients will produce the most benefit. In support of this are studies that have shown that a combination of exercise and CBT significantly reduced pain, anxiety, depression, and fatigue as well as increased physical functioning in fibromyalgia patients (Gowans et al., 1999; Mannerkorpi et al., 2000). The mechanisms of how these non-pharmacological therapies benefit chronic pain patients have not been fully established. However, in regards to exercise there is evidence that it influences both HPA axis and central sensitization pathways through altered limbic regulation and CPM respectively. CBT likely works by reducing stress in chronic pain patients, thereby decreasing HPA axis activation and downstream negative effects of this activation.

## Future Directions for the Field

Recent findings, particularly from the Multidisciplinary Approach to Pelvic Pain (MAPP) network, have greatly increased our knowledge of phenotypic and central changes that occur in patients with centralized pain syndromes (Clemens et al., 2014; Kutch et al., 2017a, b; Lai et al., 2017; Harper et al., 2018). We know that changes in gray matter density and functional connectivity are associated with widespreadness of pain and negative affect (Kutch et al., 2017a); however, we do not know what causes some patients to develop these exaggerated symptoms, while some never progress beyond localized or regional pain with no additional comorbidities. Animal models can help identify the contribution of the HPA axis, in terms of assessing whether exposure to early life adversity can functionally and structurally alter the brain, thereby increasing the susceptibility to developing centralized pain. Conversely, these types of investigations may shed light on whether adverse early life experiences result in hyperalgesic priming, thereby inducing central changes following subsequent exposure to noxious stimuli. It is clear that pain and comorbidity arise independent of symptomatic differences in cortisol release. Therefore, the dysfunction of the HPA axis, regardless of the direction it goes, may be key to the downstream dysregulation of neuroimmune interactions and increased peripheral drive. Future studies centered on restoring proper HPA axis output and regulation should heed the results of previous studies (Sweetser et al., 2009; Hubbard et al., 2011) and focus on the emotional and affective aspects of pain in patients exhibiting evidence of central nervous system pain amplification, and not just on symptomology within a broad group of patients. It has become clear that the comprehensive phenotyping of chronic pain patients will be essential for determining how centralized their pain has become, if at all, and for determining the most effective course of treatment to help ease their symptoms.

## Conclusion

Centralized pain syndromes are often comorbid with one another and are accompanied by fatigue, mood and sleep disturbances, and poor quality of life scores (Clauw, 2015). This high degree of comorbidity and a lack of definitive underlying etiology make these syndromes notoriously difficult to treat. Despite heterogeneity in presentation, these syndromes do appear to share some similar underlying mechanisms. Abnormal regulation and output of the HPA axis is commonly associated with centralized pain disorders as evidenced by altered cortisol release in patients and preclinical research in rodent models of stress-induced pain. Disrupted HPA axis output can lead to central and peripheral changes that have negative downstream consequences, including an increase in epithelial leakage and MC activation/nociceptor interaction. Increased c-fiber activation can lead to “hyperalgesic priming” and/or “wind-up” and eventually to central sensitization through LTP in the CNS. Therefore, although HPA axis dysregulation and central sensitization are separate phenomena, they share some mechanistic overlap suggesting that they could work together to cause, worsen, and/or maintain symptoms associated with centralized pain disorders. Currently, there are no established treatments for most chronic pain disorders. Non-pharmacological treatments, such as exercise and CBT, show evidence of being beneficial in the treatment of fibromyalgia, chronic pelvic pain, and migraine patients. It is likely that the most efficacious treatment for chronic pain will be a combination of treatments tailored specifically to each individual patient, based on life history and symptomology.

## Author Contributions

OCE-S, ALN and JAC: equal contribution for the literature search and article preparation. All authors contributed towards the development, research, and writing of this review.

## Conflict of Interest Statement

The authors declare that the research was conducted in the absence of any commercial or financial relationships that could be construed as a potential conflict of interest.
